# Characterizing OXPHOS inhibitor-mediated alleviation of hypoxia using high-throughput live cell-imaging

**DOI:** 10.1186/s40170-024-00342-6

**Published:** 2024-05-03

**Authors:** Anne P. M. Beerkens, Daan F. Boreel, James A. Nathan, Jiri Neuzil, Gang Cheng, Balaraman Kalyanaraman, Micael Hardy, Gosse J. Adema, Sandra Heskamp, Paul N. Span, Johan Bussink

**Affiliations:** 1 Radiotherapy & OncoImmunology Laboratory, Department of Radiation Oncology, Radboud University Medical Center, Nijmegen, 6525GA The Netherlands; 2 Department of Medical Imaging, Radboud University Medical Center, Nijmegen, 6525GA The Netherlands; 3 Department of Medicine, Cambridge Institute for Medical Research, University of Cambridge, Cambridge, CB2 0XY UK; 4 School of Pharmacy and Medical Science, Griffith University, Southport Qld, 4222 Australia; 5 Institute of Biotechnology, Czech Academy of Sciences, Prague-West, 252 50 Czech Republic; 6 Department of Biophysics, Medical College of Wisconsin, 8701 Watertown Plank Road, Milwaukee, WI 53226 USA; 7 Aix Marseille University, CNRS, ICR, UMR 7273, Marseille, 13013 France

**Keywords:** Hypoxia, Metabolic reprogramming, OXPHOS inhibitors, Mitochondria-targeting, Spheroids, Live cell imaging

## Abstract

**Background:**

Hypoxia is a common feature of many solid tumors and causes radiotherapy and immunotherapy resistance. Pharmacological inhibition of oxidative phosphorylation (OXPHOS) has emerged as a therapeutic strategy to reduce hypoxia. However, the OXPHOS inhibitors tested in clinical trials caused only moderate responses in hypoxia alleviation or trials were terminated due to dose-limiting toxicities. To improve the therapeutic benefit, FDA approved OXPHOS inhibitors (e.g. atovaquone) were conjugated to triphenylphosphonium (TPP^+^) to preferentially target cancer cell’s mitochondria. In this study, we evaluated the hypoxia reducing effects of several mitochondria-targeted OXPHOS inhibitors and compared them to non-mitochondria-targeted OXPHOS inhibitors using newly developed spheroid models for diffusion-limited hypoxia.

**Methods:**

B16OVA murine melanoma cells and MC38 murine colon cancer cells expressing a HIF-Responsive Element (HRE)-induced Green Fluorescent Protein (GFP) with an oxygen-dependent degradation domain (HRE-eGFP-ODD) were generated to assess diffusion-limited hypoxia dynamics in spheroids. Spheroids were treated with IACS-010759, atovaquone, metformin, tamoxifen or with mitochondria-targeted atovaquone (Mito-ATO), PEGylated mitochondria-targeted atovaquone (Mito-PEG-ATO) or mitochondria-targeted tamoxifen (MitoTam). Hypoxia dynamics were followed and quantified over time using the IncuCyte Zoom Live Cell-Imaging system.

**Results:**

Hypoxic cores developed in B16OVA.HRE and MC38.HRE spheroids within 24 h hours after seeding. Treatment with IACS-010759, metformin, atovaquone, Mito-PEG-ATO and MitoTam showed a dose-dependent reduction of hypoxia in both B16OVA.HRE and MC38.HRE spheroids. Mito-ATO only alleviated hypoxia in MC38.HRE spheroids while tamoxifen was not able to reduce hypoxia in any of the spheroid models. The mitochondria-targeted OXPHOS inhibitors demonstrated stronger anti-hypoxic effects compared to the non-mito-targeted OXPHOS inhibitors.

**Conclusions:**

We successfully developed a high-throughput spheroid model in which hypoxia dynamics can be quantified over time. Using this model, we showed that the mitochondria-targeted OXPHOS inhibitors Mito-ATO, Mito-PEG-ATO and MitoTam reduce hypoxia in tumor cells in a dose-dependent manner, potentially sensitizing hypoxic tumor cells for radiotherapy.

**Supplementary Information:**

The online version contains supplementary material available at 10.1186/s40170-024-00342-6.

## Introduction

Hypoxia is a common feature of many solid tumors [1]. The scarcity of oxygen is caused by limited diffusion of oxygen into tissues at further distance from blood vessels in combination with the increased oxygen consumption of tumor cells, or can be the result of chaotic vasculature leading to temporal occlusions of vessels [1–3]. Hypoxic tumor areas are highly resistant to radiotherapy and chemotherapy, and have a negative impact on immunotherapy outcome [4–6]. An important radioresistance mechanism is the reversion of radical-induced DNA damage under hypoxia, while the damage is permanently fixed in the presence of oxygen (oxygen fixation hypothesis) [7, 8].

Previous attempts to reduce hypoxia in combination with radiotherapy resulted in improved tumor control, but due to toxicity and concerns about practicality in the clinic, this never became standard of care [9]. These strategies also had no durable effect on the anticancer immune response, which is also important for the efficacy of radiotherapy and immunotherapy [10]. An emerging therapeutic strategy to alleviate hypoxia and thereby increase radiosensitivity is metabolic reprogramming of tumor cells. This can be achieved by the pharmacological inhibition of the oxidative phosphorylation (OXPHOS) to reduce the oxygen consumption rate (OCR) of tumor cells, because oxygen consumption by OXPHOS is the main cause of the occurrence of diffusion limited hypoxia [4, 9, 11]. Several OXPHOS inhibitors have been tested (as hypoxia modifier) in clinical trials. However, some potent inhibitors caused dose-limiting toxicities, while the inhibitors with established safety profiles only caused moderate responses in hypoxia alleviation [12–15]. Most of these OXPHOS inhibitors tested in clinical trials are not preferentially targeting cancer cells. For this reason, researchers conjugated the lipophilic cation triphenylphosphonium (TPP^+^) to natural products or FDA-approved drugs, such as atovaquone and metformin [16–18]. TPP^+ ^is a mitochondria-targeting moiety that enables its accumulation into the mitochondrial matrix [18]. These TPP^+ ^conjugated compounds are selectively taken up into cancer cells at much higher levels compared to healthy cells, due to the hyperpolarized mitochondrial membrane of cancer cells [19, 20]. More recently, a PEGylated form of mitochondria-targeted atovaquone (Mito-PEG-ATO) was developed, by linking polyethylene glycol (PEG) chains to mitochondria-targeted atovaquone (Mito-ATO) [21]. PEGylated drugs have several advantages such as improved solubility, increased serum half-life and stability, and reduced immunogenicity [22].

There are several well-established methods to detect hypoxia in vitro, such as immunolabeling of hypoxia probes (e.g. pimonidazole), HIF-1α protein or downstream targets of HIF-1α like glucose transporter 1 (GLUT-1), lactate dehydrogenase 5 (LDH-5) and carbonic anhydrase IX (CAIX) [23, 24]. However, these existing methods cannot be used to measure tumor cell hypoxia in real-time and often require cell fixation. A promising approach to dynamically monitor hypoxia is the use of a HIF-1α reporter consisting of a HIF-Responsive Element (HRE)-induced Green Fluorescent Protein (GFP) with an oxygen-dependent degradation domain (ODD). This HRE-eGFP-ODD reporter is induced and degraded in a HIF-1α-dependent manner [25]. The aim of this study was to evaluate and compare the anti-hypoxic effects of several OXPHOS inhibitors with respect to dose response effects and time response kinetics. To investigate the effect on hypoxia, we developed a spheroid model using the HRE-eGFP-ODD construct to quantify diffusion-limited hypoxia over time. In comparison to existing models such as hypoxic chambers, this model is dependent on the oxygen consumption of tumor cells and allows us to perform high-throughput analysis by quantifying eGFP autofluorescence over time in the IncuCyte Zoom.

## Methods

### Cell culture

Ovalbumin (OVA)- transfected B16F10 (B16OVA) murine melanoma cells (provided by Dr. K.L. Rock, Dana-Farber Cancer Institute, Boston) [26] were cultured in Minimum Essential Medium (MEM; Gibco) supplemented with 5% fetal calf serum (FCS; Sigma-Aldrich), 2% sodium bicarbonate, 1.5% MEM vitamins, 1% nonessential amino acids, 1% sodium pyruvate, 1% antibiotic/antimyotic, 0.1% β-mercaptoethanol, 1 mg/mL G418 (Geneticin) (all Gibco) and 60 µg/mL hygromycin (Invitrogen). MC38 murine colon carcinoma cells (Kerafast) were cultured in Dulbecco’s Modified Eagle Medium (DMEM) with Glutamax (Gibco) supplemented with 10% FCS, 1% sodium pyruvate, 1% nonessential amino acids and 1% penicillin-streptomycin. Cell lines were incubated at 37 °C in 5% CO_2_. From the B16OVA and MC38 parental cell lines, clones were generated containing an HRE-eGFP-ODD construct (see ‘Generation of HRE-eGFP-ODD cell lines’). These clones (B16OVA.HRE-eGFP-ODD.1B8 and MC38.HRE-eGFP-ODD.2F8) were cultured under the same conditions as the parental cell lines. Mycoplasma testing was performed routinely for these cell lines using a mycoplasma detection kit (Lonza).

### Generation of HRE-eGFP-ODD cell lines

B16OVA and MC38 cells were lentivirally transduced with an HRE-eGFP-ODD construct [25]. Lentiviral particles were produced by transfection of 293FT cells with the HRE-ODD-GFP plasmid (#171) and the packaging plasmids (pLP1 gag, pLP2 rev and PLP/VSVG). Transfection was performed with cells at 90% confluency using Metafectene (Biontex Laboratories GmbH). After 24 h, medium was refreshed with culture medium of the target cells. Viral supernatant was harvested 48 h after refreshing the medium and filtered through a 45 μm filter. Subsequently, viral supernatant was added to the target cells and incubated at 37 °C for 48 h. Cells were expanded and subsequently cultured under hypoxic conditions of 1% O_2_ for 24–48 h in a Whitley H35 Workstation (Don Whitley Scientific) to induce eGFP expression. After 48 h in the hypoxic chamber, eGFP positive cells were single cell sorted using the FACS ARIA II SORP Flow Cytometer Cell Sorter (BD Biosciences). Flow cytometry analyses were performed to determine the percentage of eGFP^+ ^cells. Clone B16OVA.HRE-eGFP-ODD.1B8 (B16OVA.HRE) and clone MC38.HRE-eGFP-ODD.2F8 (MC38.HRE) were selected for subsequent experiments.

### Flow cytometry

To determine eGFP expression after incubation in the hypoxic chamber, cells were first washed with PBS and stained with eFluor 780 viability dye (eBioscience, 65-0865-14) for 20 min at 4 °C. Next, cells were washed with PBS and subsequently with PBA (0.5% bovine serum albumin in PBS). Cells were acquired and fluorescence was measured using a FACS Canto II Flow Cytometer (BD Bioscience). Data were analyzed using FlowJo software (version V10.7).

### Spheroid formation

Spheroids were formed by plating 10.000 cells/well in ultra low attachment (ULA) plates (Corning, 7007) with 2.5% Matrigel (Corning, 354234). Plates were centrifuged at 1000 rpm for 10 min at room temperature. Subsequently, the spheroids were left to form and cultured at 37 °C in 5% CO_2_.

### Live cell imaging

The IncuCyte Zoom Live-Cell Analysis System (Essen Bioscience) was used to monitor the HRE-eGFP-ODD signal in spheroids over time. The spheroids were photographed at 1 h intervals using a 4x objective. Combined phase-contrast and fluorescent images were collected for each time point to determine the amount of hypoxia per spheroid. The IncuCyte analyzer provides the Total Hypoxia Integrated Intensity (the mean fluorescent intensity (in Green Calibrated Units; GCU) of the total hypoxic area multiplied by the size of the total hypoxic area (in µm^2^)) based on the settings in the processing definition. For the green channel the following settings were applied: the parameter was set to adaptive, threshold adjustment (GCU) 4.000, Adjust Size (pixel) -1 and minimum area (µm^2^) to 30.000. The Total Hypoxia Integrated Intensity was used to calculate the relative total hypoxia integrated intensity. Only cells that adequately formed spheroids of equal sizes were analyzed. To determine the maximal half-life of the fusion protein, for each OXPHOS inhibitor (at the highest concentration) the total hypoxia integrated intensity at t = 24 h (last scan before treatment) was set to 100%. Thereby, the time at which 50% of the HRE-eGFP-ODD signal remained was determined. This process would include both inhibitor effectivity and subsequent ODD-mediated protein degradation, and therefore denotes the maximal half-life of the GFP-ODD protein.

### OXPHOS inhibitor treatment

B16OVA.HRE and MC38.HRE spheroids were treated with OXPHOS inhibitors 24 h after seeding. The following OXPHOS inhibitors were used for treatment: IACS-010759 (1, 0.33, 0.11 and 0.037 µM, Selleckchem), metformin (9, 3, 1 and 0.33 mM, Sigma-Aldrich), atovaquone (30, 15, 7.5 and 3.75 µM, Sigma-Aldrich), mito_10_-atovaquone (30, 15, 7.5 and 3.75 µM) [16], Mito-PEG_5_-Atovaquone (30, 15, 7.5 and 3.75 µM) [21], tamoxifen (5, 2.5, 1.25 and 0.625 µM, Sigma-Aldrich (H7904)) and MitoTam (5, 2.5, 1.25 and 0.625 µM) [27, 28]. Control spheroids were treated with 0.2% DMSO.

### Statistical analyses

Statistical analyses were performed using GraphPad Prism (version 9.5.0). For comparison between groups a Kruskal-Wallis test with multiple comparisons was performed. A *p*-value of < 0.05 was considered significant (*P* < 0.05 *, *P* < 0.01 **, *P* < 0.001 ***, *P* < 0.0001 ****).

## Results

### Generation of B16OVA and MC38 cells expressing an HRE-eGFP-ODD reporter

**Fig. 1:**
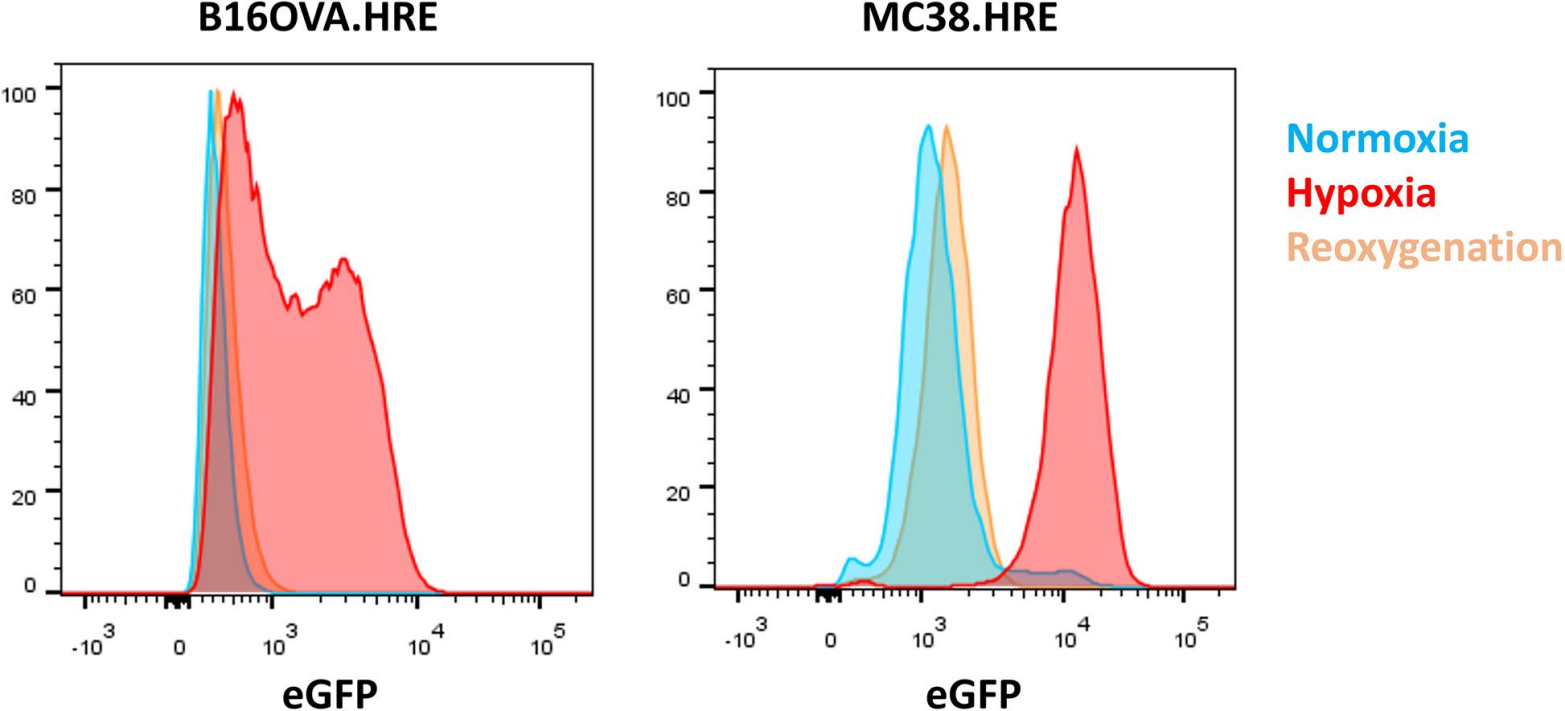
Generation of B16OVA and MC38 cells expressing a HIF-Responsive Element (HRE)-induced Green Fluorescent Protein (GFP). eGFP levels measured by flow cytometry of B16OVA and MC38 cells expressing the HRE-eGFP-ODD construct incubated in 20% oxygen (normoxia) or 1% oxygen (hypoxia) for 48 h or incubated in 1% oxygen for 24 h followed by re-exposure to 20% oxygen for 24 h (reoxygenation)

To study hypoxia dynamics, we transduced a HIF-Responsive Element (HRE)-eGFP-ODD construct into B16OVA and MC38 cells. Under hypoxia (< 2–3% O_2_), HIF-1α will bind to the HRE promoter of the transduced construct, leading to eGFP activation visible as a green fluorescent signal. Under (subsequent) normoxic conditions, GFP is rapidly degraded via the oxygen-dependent degradation domain (ODD). The B16OVA and MC38 cells transduced with the construct were sorted to select the clones with the highest expression of eGFP after incubation under hypoxic conditions. These clones also showed degradation of GFP after re-exposure to 20% oxygen (Fig. 1). Over time, the clones remain stable in their expression of HRE-eGFP-ODD under hypoxia.

### Diffusion-limited hypoxia in spheroids

**Fig. 2:**
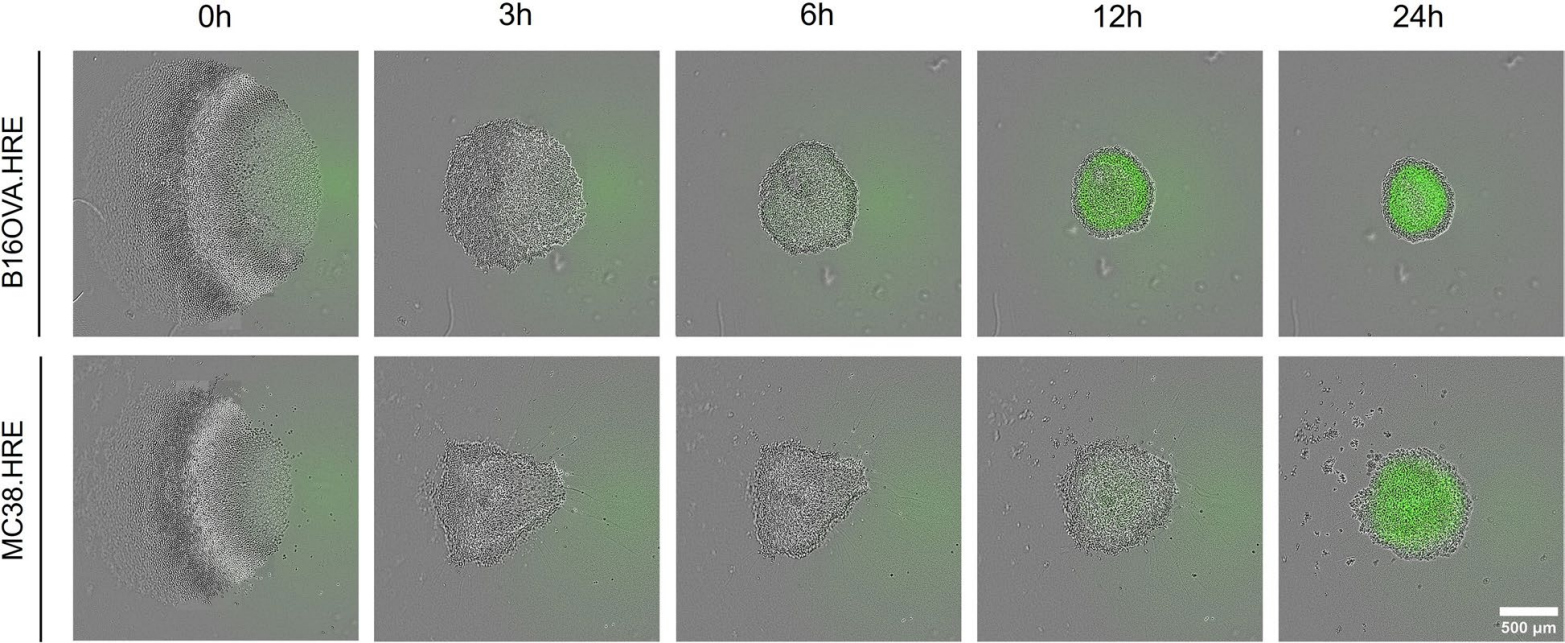
Hypoxic cores develop in B16OVA.HRE and MC38.HRE spheroids. Spheroids formed from B16OVA and MC38 cells transduced with an HRE-eGFP-ODD construct induce eGFP under hypoxia over time as detected by the IncuCyte Live-cell imaging system

To investigate and quantify diffusion-limited hypoxia, spheroids were formed from the stably transduced HRE-eGFP-ODD tumor cells and the spheroids were monitored over time by live imaging using the IncuCyte Zoom system. Spheroids were grown at sizes known to induce hypoxic cores, because of diffusion limits under normoxic conditions [29]. In our B16OVA.HRE and MC38.HRE spheroid models, hypoxic cores were readily detected within 24 h after spheroid formation (Fig. 2). The IncuCyte Zoom System allows us to monitor up to six 96-wells plates at the same time. So, using these in vitro models, we are able to monitor and quantify diffusion-limited hypoxia in spheroids over time in a high-throughput manner.

### Metabolic reprogramming in hypoxic spheroids

The dynamic high-throughput spheroid model described above was used to evaluate the anti-hypoxic effects of several OXPHOS inhibitors. Some of these OXPHOS inhibitors have already been tested (as hypoxia modifiers) in clinical trials, while others are still in preclinical research (Table 1).

**Table 1:** List of OXPHOS inhibitors tested in this study

OXPHOS inhibitor	Mitochondrial complex targeted	Selected clinical trials as anticancer therapeutic	Tested in selected trial as hypoxia modifier	Purpose	Remarks
IACS-010759	I	NCT02882321	No	Phase I, assess safety and efficacy in advanced solid tumors and acute myeloid leukemia	Both trials discontinued due to toxicities
		NCT03291938	No		
Metformin	I	NCT02394652	Yes	Phase II, window-of-opportunity trial in locally advanced cervical cancer	
Atovaquone	III	NCT0268080	Yes	Phase I, window-of-opportunity trial in NSCLC	
		NCT04648033	Yes	Phase I, determine MTD of ATO in combination with concurrent CRT in NSCLC	
Mito-Atovaquone	I, III	-	-		Pre-clinical study: anti-tumor effect of In Situ vaccination with Mito-ATO
Mito-PEG-Atovaquone	I, III	-	-	-	
Tamoxifen	I, II, III, IV	NCT03280563	No	Phase II, assess safety, efficacy and pharmacokinetics of anti-PD-L1 mAb also combined with tamoxifen in metastatic breast cancer	
		NCT00003857	No	Phase III, effectiveness of radiotherapy with or without Tamoxifen in ductal carcinoma in situ	
MitoTam	I	MitoTam-01, EudraCt 2017-004441-25	No	Phase I/Ib, evaluate drug safety and MTD, and long term toxicity	

*Abbreviations: NSCLC *Non-small Cell Lung Carcinoma*, MTD* Maximum tolerated dose*,* *CRT* *C*hemoradiotherapy

**Fig. 3:**
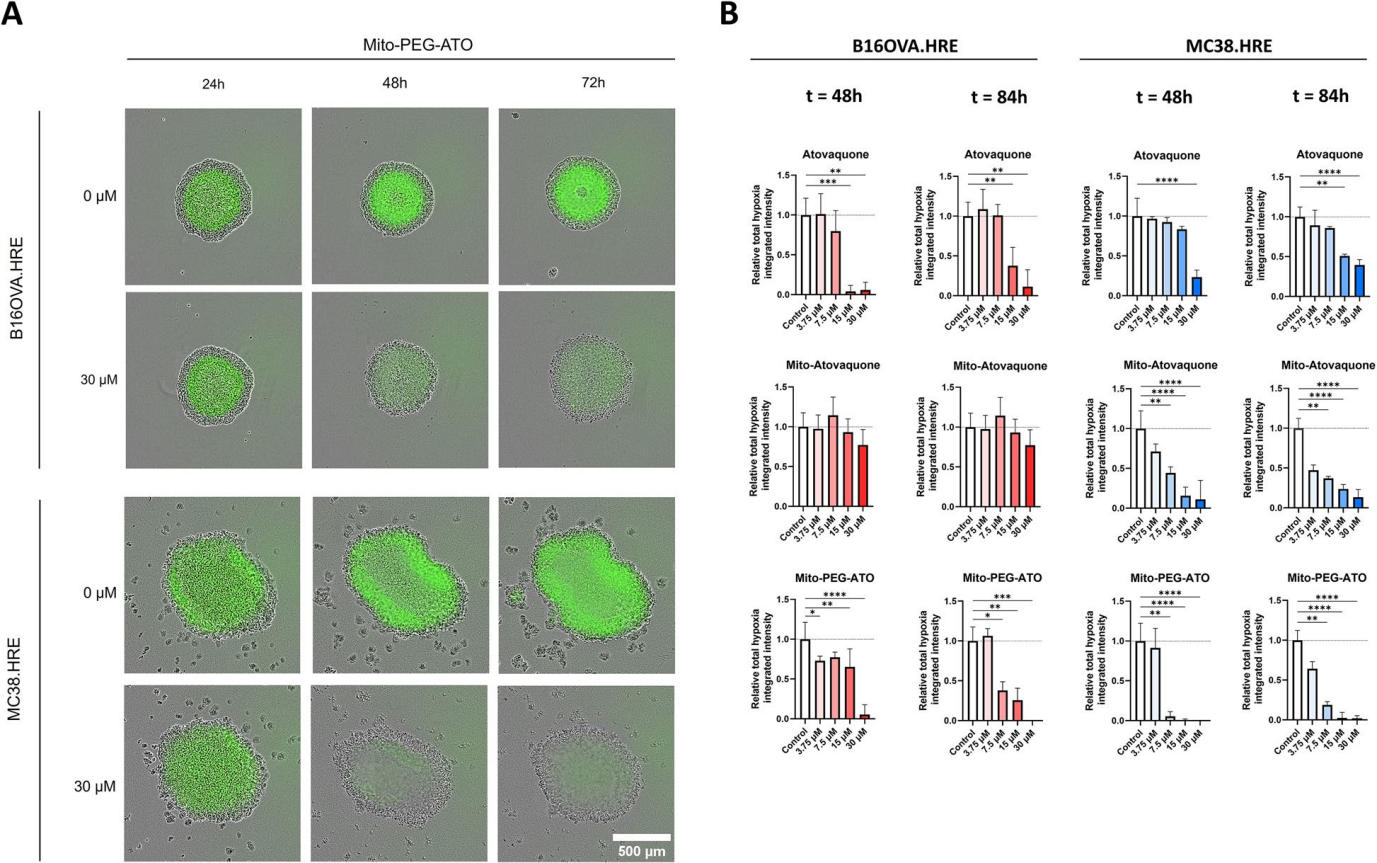
OXPHOS inhibitors alleviate diffusion-limited hypoxia in a dose-dependent manner in spheroids. **A** The effect of Mito-PEG-ATO on hypoxia in B16OVA.HRE and MC38.HRE spheroids was monitored in the IncuCyte Live-cell imaging system. Spheroids were treated with 30 µM Mito-PEG-ATO or with 0.2% DMSO (control) 24 h after spheroid formation. **B** Quantification of the total hypoxia integrated intensity (the mean fluorescent intensity of the total hypoxic area multiplied by the size of the total hypoxic area) in B16OVA.HRE (red graphs) and MC38.HRE (blue graphs) spheroids compared to controls. T = 0 indicates the start of the formation of B16OVA.HRE and MC38.HRE spheroids. At t = 24, spheroids were treated with atovaquone, mito-atovaquone, PEGylated mito-atovaquone (Mito-PEG-ATO) or 0.2% DMSO (control). Data are presented as mean with SD, *n* ≥ 5

The total amount of hypoxia (total hypoxia integrated intensity) per spheroid was quantified to evaluate and compare the anti-hypoxic effects of IACS-010759, metformin, atovaquone, Mito-ATO, Mito-PEG-ATO, tamoxifen and MitoTam. The images retrieved from the Incucyte showed an almost complete alleviation of diffusion-limited hypoxia within 24 h after treatment with 30 µM Mito-PEG-ATO in both B16OVA.HRE and MC38.HRE spheroids. In contrast, there was no reduction of hypoxia detected in the control spheroids (Fig. 3A). These images of spheroids treated with the different OXPHOS inhibitors or control were used to quantify the total amount of hypoxia per spheroid. Quantification of hypoxia showed that compared to the control, B16OVA.HRE spheroids treated with atovaquone (15 µM, 30 µM) resulted in a significant reduction of hypoxia 24 h after treatment (t = 48 h) (*P* = 0.0002 and *P* = 0.0012, respectively). This anti-hypoxic effect was still present 60 h after treatment (t = 84 h) for the two highest concentrations. In MC38.HRE spheroids, hypoxia was also alleviated in a dose-dependent manner after treatment with atovaquone. The anti-hypoxic effect was stronger at a later timepoint after treatment (t = 84 h), where spheroids treated with 15 µM and 30 µM atovaquone showed a significant reduction in hypoxia of at least 50% (*P* = 0.0013 and *P* < 0.0001, respectively) (Fig. 3B). Similar results were observed after treatment with IACS-010759 and metformin in B16OVA.HRE and MC38.HRE spheroids. Both OXPHOS inhibitors also caused a dose-dependent reduction in hypoxia, which persisted for several days. In contrast, B16OVA.HRE and MC38.HRE spheroids treated with tamoxifen did not show a significant alleviation of hypoxia (Supplementary Fig. 1).

Next, we quantified the hypoxia reducing effects of the mitochondria-targeted OXPHOS inhibitors Mito-ATO, Mito-PEG-ATO and MitoTam. Interestingly, treatment with Mito-ATO only resulted in a significant alleviation of hypoxia in MC38.HRE spheroids and not in B16OVA.HRE spheroids. MC38.HRE spheroids treated with Mito-ATO showed a significant dose-dependent alleviation of hypoxia at both timepoints. Surprisingly, treatment with Mito-PEG-ATO significantly decreased hypoxia 24 h after administration (t = 48 h) in B16OVA.HRE spheroids. This effect was even stronger 60 h after treatment (t = 84 h), showing a reduction of more than 50% after treatment with at least 7.5 µM Mito-PEG-ATO. In addition, MC38.HRE spheroids treated with at least 7.5 µM Mito-PEG-ATO demonstrated a significant reduction of hypoxia at both timepoints. The anti-hypoxic effect was strongest after treatment with 15 µM and 30 µM of Mito-PEG-ATO, showing an almost complete response at both timepoints (all *P* < 0.0001) (Fig. 3B). Furthermore, treatment with MitoTam also resulted in a significant alleviation of hypoxia in both B16OVA.HRE and MC38.HRE spheroids (Supplementary Fig. 1).

Comparing the mitochondria-targeted inhibitors to the non-mitochondria-targeted inhibitors, we observed that IACS-010759, metformin and atovaquone alleviate hypoxia within 12 h after treatment, while for the mitochondria-targeted inhibitors Mito-ATO, Mito-PEG-ATO and MitoTam the maximum reduction is around 24 h after treatment (Supplementary Fig. 2). To elaborate more on the effect rate between the different OXPHOS inhibitors, we calculated the time at which 50% of the HRE-eGFP-ODD signal was reduced after treatment with each OXPHOS inhibitor (Supplementary Table 1). Hypoxia is reduced with 50% within 2 h after treatment with IACS-010759 and atovaquone, while for metformin and the mito-targeted inhibitors this takes longer and ranges between 7.0 and 28.5 h in B16OVA spheroids and between 2.8 and 13.0 h in MC38 spheroids. These results also showed that the half-life of HRE-eGFP-ODD is shorter than 0.8 h, as this was the fastest reduction seen after treatment of MC38 spheroids with IACS-010759. In addition, the hypoxia reducing effect of the OXPHOS inhibitors Mito-ATO, Mito-PEG-ATO, MitoTam, IACS-010759 and metformin was stronger in MC38.HRE spheroids compared to B16OVA.HRE spheroids.

Altogether, IACS-010759, metformin, atovaquone, Mito-PEG-ATO and MitoTam showed a dose-dependent alleviation of hypoxia after treatment in both B16OVA.HRE and MC38.HRE spheroids. The mitochondria-targeted inhibitors demonstrated stronger anti-hypoxic effects compared to their untargeted analogs, except for treatment of Mito-ATO in B16OVA.HRE spheroids. The anti-hypoxic effect was strongest after treatment with Mito-PEG-ATO and the effect lasted for several days.

## Discussion

Here, we applied a high-throughput live cell imaging model to quantify diffusion-limited hypoxia over time. Using this model, we found a differential effect of mitochondria-targeting OXPHOS inhibitors on hypoxia alleviation compared to non-mitochondria-targeted compounds. The mitochondria-targeted OXPHOS inhibitors Mito-ATO, Mito-PEG-ATO and MitoTam demonstrated stronger anti-hypoxic effects compared to non-targeted atovaquone and tamoxifen. We also found that for Mito-ATO, Mito-PEG-ATO and MitoTam it took longer before hypoxia was reduced. Inhibition of OXPHOS to reduce the oxygen consumption rate of tumor cells has emerged as a promising strategy to alleviate hypoxia over prolonged periods of time and thereby possibly improving radiotherapy and immunotherapy outcome [6, 11, 30]. A short lasting increase in the tumor oxygenation status at the time of irradiation resulted in improved tumor control (hyperbaric oxygen and ARCON) [31, 32]. However, our strategy using OXPHOS inhibitors caused a durable response and therefore possibly has a sustained effect on the local immune cell repertoire.

There are several methods to detect hypoxia, however there is no model in which hypoxia dynamics can be quantified in tumor equivalents in real time. Therefore, we developed two in vitro spheroid models containing an HRE-eGFP-ODD construct [25], which allowed us to evaluate the anti-hypoxic effects of several OXPHOS inhibitors and to compare their potential as hypoxia modifier. Treatment with IACS-010759, metformin and atovaquone caused a dose-dependent reduction in hypoxia in our spheroid models. In another study from our group we showed that IACS-010759, atovaquone and metformin reduce the OCR of B16OVA and MC38 cells, indicating the correlation between hypoxia decrease in spheroids and decreased oxygen consumption rate in monolayers [33]. This is in line with previous studies showing clinical evidence that tumor hypoxia can be reduced using metformin and atovaquone [12, 13]. The highly potent mitochondrial complex I inhibitor IACS-010759 showed promising anti-cancer effects in pre-clinical studies [34–36]. However, two independent phase I clinical trials were prematurely terminated because of unacceptable dose-limiting toxicities including neurotoxicity and elevated blood lactate levels [14], indicating the potency of this OXPHOS inhibitor but also the very narrow therapeutic window. A possible explanation for the small therapeutic window of IACS-010759 is that this OXPHOS inhibitor is not preferentially targeting cancer cells and thus may cause off-target tissue toxicity. Currently, two phase I trials are ongoing testing another mitochondrial complex I inhibitor, the phosphodiesterase inhibitor papaverine, in combination with stereotactic body radiation therapy or chemoradiation (NCT03824327, NCT05136846). This inhibitor improved tumor oxygenation in mouse tumor models and canine patients [37, 38]. In the clinical trials using the moderate OXPHOS inhibitors atovaquone and metformin not all patients responded, especially in the metformin trial only half of the patients showed a reduction in hypoxia and in several patients only a small reduction was visible [12, 13]. This reduction in hypoxia might be too small to have a sustained effect on the radiotherapy and immunotherapy efficacy. Therefore, more potent hypoxic radiosensitizers with a wider therapeutic window are needed, thereby limiting off-target tissue toxicity.

In contrast to IACS-010759, metformin and atovaquone, tamoxifen was not able to alleviate hypoxia in our spheroid models. For a long time, tamoxifen has been considered, besides being an estrogen receptor inhibitor, as a compound able to exhibit OXPHOS inhibitory effects by targeting several mitochondrial complexes [39]. However, these studies applied fragments of isolated mitochondria, and recently it was shown that tamoxifen is not able to penetrate across the mitochondrial membrane of intact mitochondria, and therefore is unable to inhibit OXPHOS in living cells [40]. These findings explain why tamoxifen was not able to reduce hypoxia in our studies with B16OVA.HRE and MC38.HRE spheroids. This is also in line with another study which demonstrated that the OCR of MCF7 breast cancer cells did not change after acute tamoxifen treatment [41].

Although tamoxifen does not appear to be an OXPHOS inhibitor, MitoTam is able to inhibit OXPHOS in B16OVA.HRE and MC38.HRE spheroids leading to hypoxia relief. This difference compared to tamoxifen can be explained by the fact that MitoTam is able to penetrate the mitochondrial membrane. The TPP^+^ conjugation of MitoTam is neutralized thereby making the penetration into the mitochondria favorable. In contrast, the localized positive charge of tamoxifen could be unfavorable, preventing tamoxifen from entering the mitochondria [40].

While treatment with Mito-PEG-ATO causes a near complete loss of hypoxia in both B16OVA.HRE and MC38.HRE spheroids, a reduction in hypoxia was only observed in MC38.HRE spheroids after treatment with Mito-ATO. In addition, non-mito-targeted atovaquone also caused a dose-dependent alleviation in hypoxia in both spheroid models. The differential effects in hypoxia relief between Mito-ATO and Mito-PEG-ATO are most likely caused by the much higher hydrophobicity of the former. PEGylation increases side chain length without a significant increase in hydrophobicity, so the hydrophobicity of Mito-PEG-ATO is lower compared to Mito-ATO [21]. This in combination with differences in mitochondrial membrane potential and mitochondrial membrane composition could explain the difference in uptake of these drugs in the two spheroids models. Also, in general we observed that the anti-hypoxic effect of the tested OXPHOS inhibitors was stronger in MC38.HRE spheroids compared to B16OVA.HRE spheroids, suggesting differences in the levels of OXPHOS genes between those two cell lines. This is in line with the results from another study from our group, showing that the basal OCR rate of MC38 cells is higher compared to the basal OCR rate of B16OVA cells [33].

Comparing the mito-targeted inhibitors to the non-mito-targeted inhibitors we observed that the non-mito-targeted inhibitors IACS-010759 and atovaquone work faster, as they reach a 50% reduction in hypoxia earlier compared to the mito-targeted compounds. These differences are probably caused by how easily the inhibitors penetrate the cell and mitochondrial membrane [42]. Mito-PEG-ATO treatment caused the strongest anti-hypoxic effect of all OXPHOS inhibitors tested in this study. The anti-hypoxic effect of Mito-PEG-ATO was even stronger than the effect of the potent small molecule inhibitor IACS-010759. A possible explanation for this might be that Mito-PEG-ATO, as well as Mito-ATO, inhibit both mitochondrial complex I and III, while atovaquone only inhibits complex III and IACS-010759 only complex I [16, 21, 34, 43]. The length of the side chain of Mito-ATO and Mito-PEG-ATO influences their mitochondrial targeting and oxygen consumption inhibition. The analogs of mito-atovaquone (Mito_10_ATO) and PEGylated mito-atovaquone (Mito-PEG_5_-ATO) we used in this study selectively inhibit both mitochondrial complex I and III [16, 21].

In this study, we investigated the anti-hypoxic effects of Mito-ATO, Mito-PEG-ATO and MitoTam. Earlier d’Hose et al. examined whether treatment with mitochondria-targeted metformin (mito-metformin) can radiosensitize prostate cancer cells in vivo. Although the amount of hypoxia significantly decreased upon treatment with mito-metformin, it had no additive effect on radiotherapy efficacy [44]. The effect of Mito-ATO on hypoxia alleviation has not been studied previously. However, the effect of Mito-ATO on immune cells in the tumor microenvironment of mouse tumor models has been investigated. In situ vaccination with Mito-ATO resulted in inhibition of tumor growth in both transplanted and spontaneous tumor models. Interestingly, Mito-ATO caused a significant reduction in regulatory T cells and granulocytic myeloid-derived suppressor cells, and an increase in infiltrating CD4 + T cells. In addition, Mito-ATO combined with anti-PD1 therapy resulted in a significant survival advantage in mice compared to the controls [45]. Similar studies should be performed to investigate the effect of Mito-PEG-ATO on immune cells and the immunogenicity, because PEG can be both immunogenic and antigenic according to literature [46].

We showed the relevance of our developed spheroid model to test the effect of several OXPHOS on diffusion-limited hypoxia. Compared to 2D cell cultures this is an important advantage, because in our spheroids we can monitor hypoxia caused by the oxygen consumption of tumor cells instead of incubating cells under hypoxic conditions in 2D. However, our spheroid model does not contain a whole tumor microenvironment including for instance immune cells or cancer associated fibroblasts. In addition, in a spheroid we cannot test whether the mito-targeted inhibitors preferentially target the mitochondria of cancer cells rather than the mitochondria of healthy cells. Therefore, future studies are needed to elucidate whether Mito-ATO, Mito-PEG-ATO and MitoTam are able to alleviate hypoxia in vivo sufficiently to radiosensitize cancer cells.

## Conclusions

We developed a high-throughput model in which diffusion-limited hypoxia can be quantified over time. Using this model, we demonstrated that the mitochondria-targeted compounds Mito-ATO, Mito-PEG-ATO and MitoTam alleviate hypoxia in a dose-dependent manner, which could sensitize hypoxic tumor cells for radiotherapy, thereby increasing the therapeutic window. Pre-clinical studies are required to determine whether Mito-ATO, Mito-PEG-ATO and MitoTam are able to modify the radioresistant and immune suppressive tumor microenvironment to a radiosensitive and immune permissive environment.

### Supplementary Information

**Additional file 1: Supplementary Fig. 1.** Effect of IACS-010759, metformin, tamoxifen and MitoTam on diffusion-limited hypoxia in spheroids. Quantification of the total hypoxia integrated intensity (the mean fluorescent intensity of the total hypoxic area multiplied by the size of the total hypoxic area) in B16OVA.HRE (red graphs) and MC38.HRE (blue graphs) spheroids compared to controls. T = 0 indicates the start of the formation of B16OVA.HRE and MC38.HRE spheroids. At t = 24, spheroids were treated with IACS-010759, metformin, tamoxifen, MitoTam or 0.2% DMSO (control). Data are presented as mean with SD, *n* ≥ 5.

**Additional file 2: Supplementary Fig. 2.** Effect of several OXPHOS inhibitors on diffusion-limited hypoxia over time in spheroids. Quantification of the total hypoxia integrated intensity (the mean fluorescent intensity (in GCU) of the total hypoxic area multiplied by the size of the total hypoxic area (in µm^2^)) over time. T = 0 indicates the start of the formation of B16OVA.HRE and MC38.HRE spheroids. At t = 24, spheroids were treated with IACS-010759, (IACS), metformin (MET), atovaquone (ATO), mito-atovaquone (Mito-ATO), PEGylated mito-atovaquone (Mito-PEG-ATO), tamoxifen, MitoTam or 0.2% DMSO (control). Data are presented as mean, *n* ≥ 5. GCU = Green Calibrated Units.

**Additional file 3: Supplementary Table 1.** Effect rate of several OXPHOS inhibitors in B16OVA.HRE and MC38.HRE spheroids. Time at which the HRE-eGFP-ODD signal is reduced by 50% after treatment with several OXPHOS inhibitors in B16OVA.HRE and MC38.HRE spheroids.

## Data Availability

No datasets were generated or analysed during the current study.

## Abbreviations

OXPHOS: Oxidative phosphorylation
OCR: Oxygen consumption rate
TPP^+^: Triphenylphosphonium
Mito-atovaquone, Mito-ATO: Mitochondria-targeted atovaquone
Mito-PEG-ATO: PEGylated mito-atovaquone
PEG: Polyethylene glycol
HRE-eGFP-ODD: HIF-responsive element (HRE)-induced green fluorescent protein (GFP) with oxygen degradation domain (ODD)
OVA: Ovalbumin
B16OVA.HRE: B16OVA.HRE-eGFP-ODD.1B8
MC38.HRE: MC38.HRE-eGFP-ODD.2F8
GCU: Green Calibrated Units
MitoTam: Mitochondria-targeted Tamoxifen
NSCLC: Non-small Cell Lung Carcinoma
MTD: Maximum tolerated dose
CRT: Chemoradiotherapy
