# Supplementary figures and images for: Characterizing OXPHOS inhibitor-mediated alleviation of hypoxia using high-throughput live cell-imaging
Source: Cancer Metab. 2024 May 3;12:13. doi: 10.1186/s40170-024-00342-6 (PMC11067257; doi:10.1186/s40170-024-00342-6)

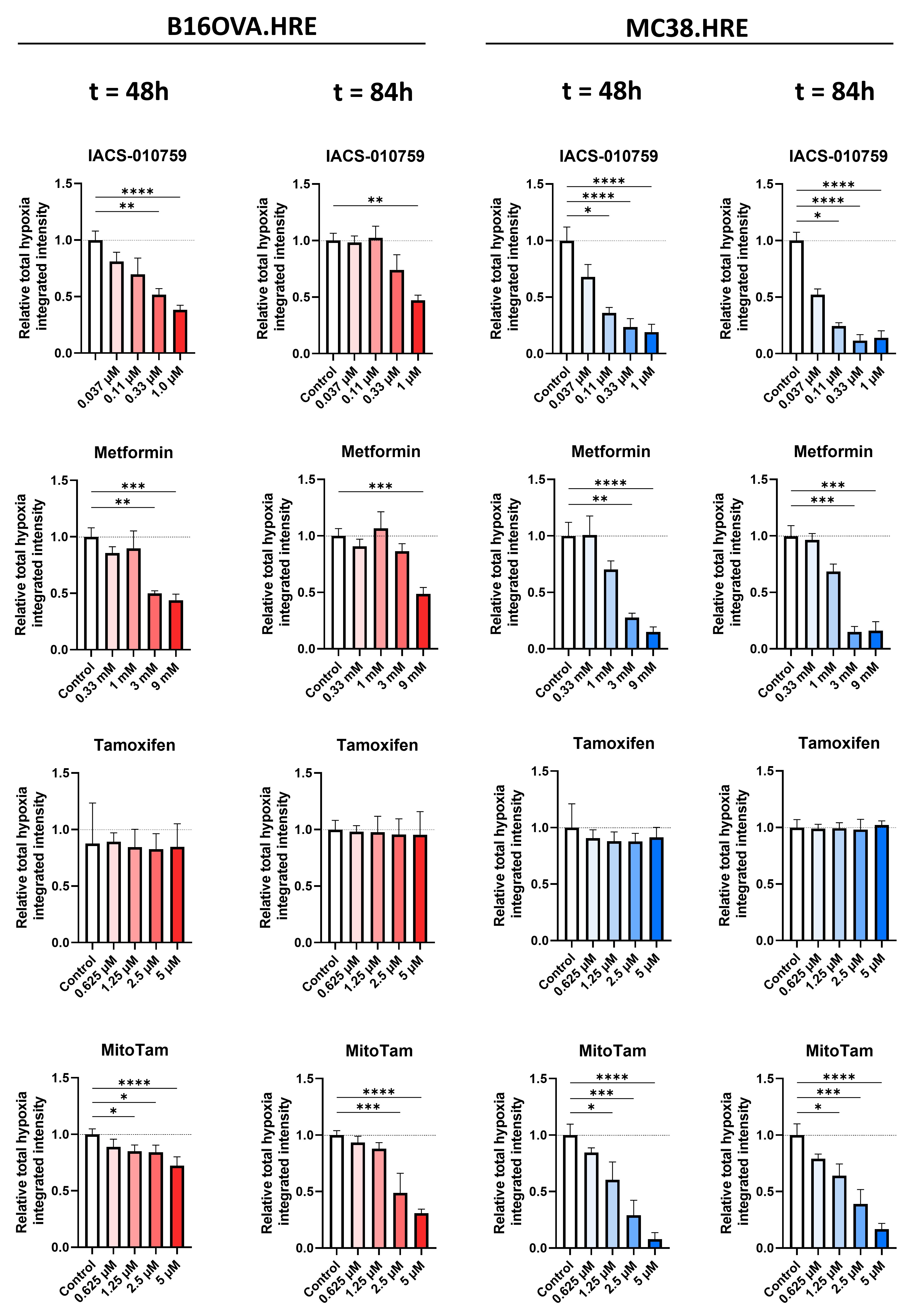

Supplement: Supplementary file 1 — Additional file 1: Supplementary Fig. 1. Effect of IACS-010759, metformin, tamoxifen and MitoTam on diffusion-limited hypoxia in spheroids. Quantification of the total hypoxia integrated intensity (the mean fluorescent intensity of the total hypoxic area multiplied by the size of the total hypoxic area) in B16OVA.HRE (red graphs) and MC38.HRE (blue graphs) spheroids compared to controls. T = 0 indicates the start of the formation of B16OVA.HRE and MC38.HRE spheroids. At t = 24, spheroids were treated with IACS-010759, metformin, tamoxifen, MitoTam or 0.2% DMSO (control). Data are presented as mean with SD, n ≥ 5. [file 40170_2024_342_MOESM1_ESM.jpg]

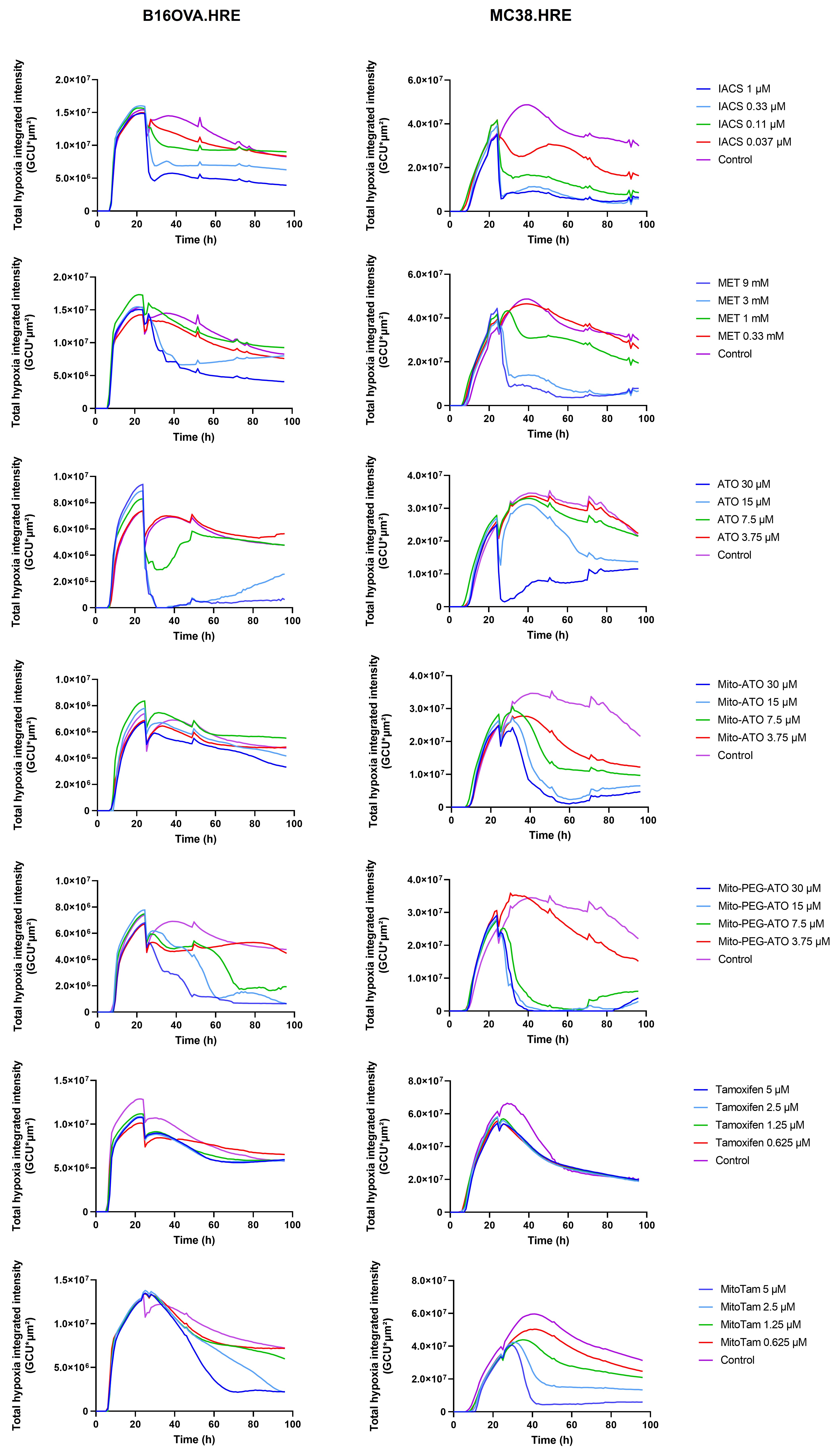

Supplement: Supplementary file 2 — Additional file 2: Supplementary Fig. 2. Effect of several OXPHOS inhibitors on diffusion-limited hypoxia over time in spheroids. Quantification of the total hypoxia integrated intensity (the mean fluorescent intensity (in GCU) of the total hypoxic area multiplied by the size of the total hypoxic area (in µm2)) over time. T = 0 indicates the start of the formation of B16OVA.HRE and MC38.HRE spheroids. At t = 24, spheroids were treated with IACS-010759, (IACS), metformin (MET), atovaquone (ATO), mito-atovaquone (Mito-ATO), PEGylated mito-atovaquone (Mito-PEG-ATO), tamoxifen, MitoTam or 0.2% DMSO (control). Data are presented as mean, n ≥ 5. GCU = Green Calibrated Units. [file 40170_2024_342_MOESM2_ESM.jpg]

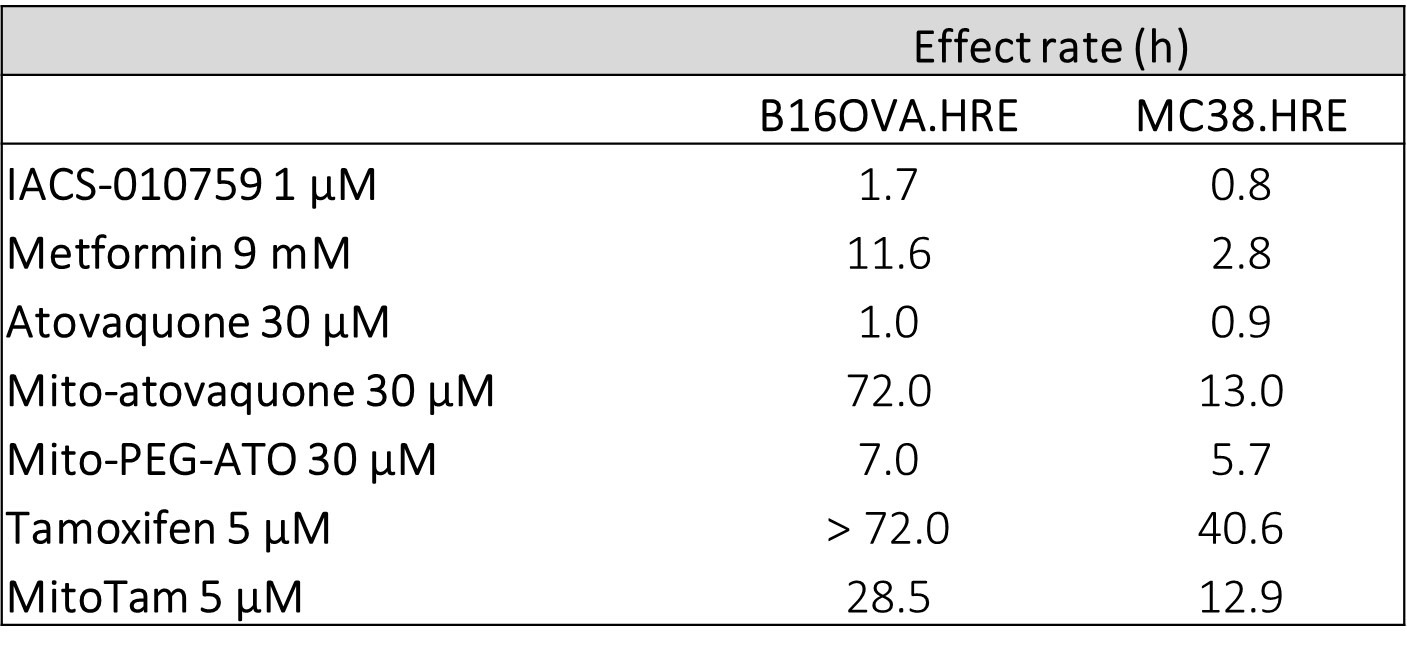

Supplement: Supplementary file 3 — Additional file 3: Supplementary Table 1. Effect rate of several OXPHOS inhibitors in B16OVA.HRE and MC38.HRE spheroids. Time at which the HRE-eGFP-ODD signal is reduced by 50% after treatment with several OXPHOS inhibitors in B16OVA.HRE and MC38.HRE spheroids. [file 40170_2024_342_MOESM3_ESM.jpg]
